# Supplementary material for: In Utero Exposure to Persistent Organic Pollutants and Childhood Lipid Levels
Source: Metabolites. 2021 Sep 28;11(10):657. doi: 10.3390/metabo11100657 (PMC8540619; doi:10.3390/metabo11100657)
Supplement: Supplementary file 1 [file metabolites-11-00657-s001.zip › Figure S1.pdf]

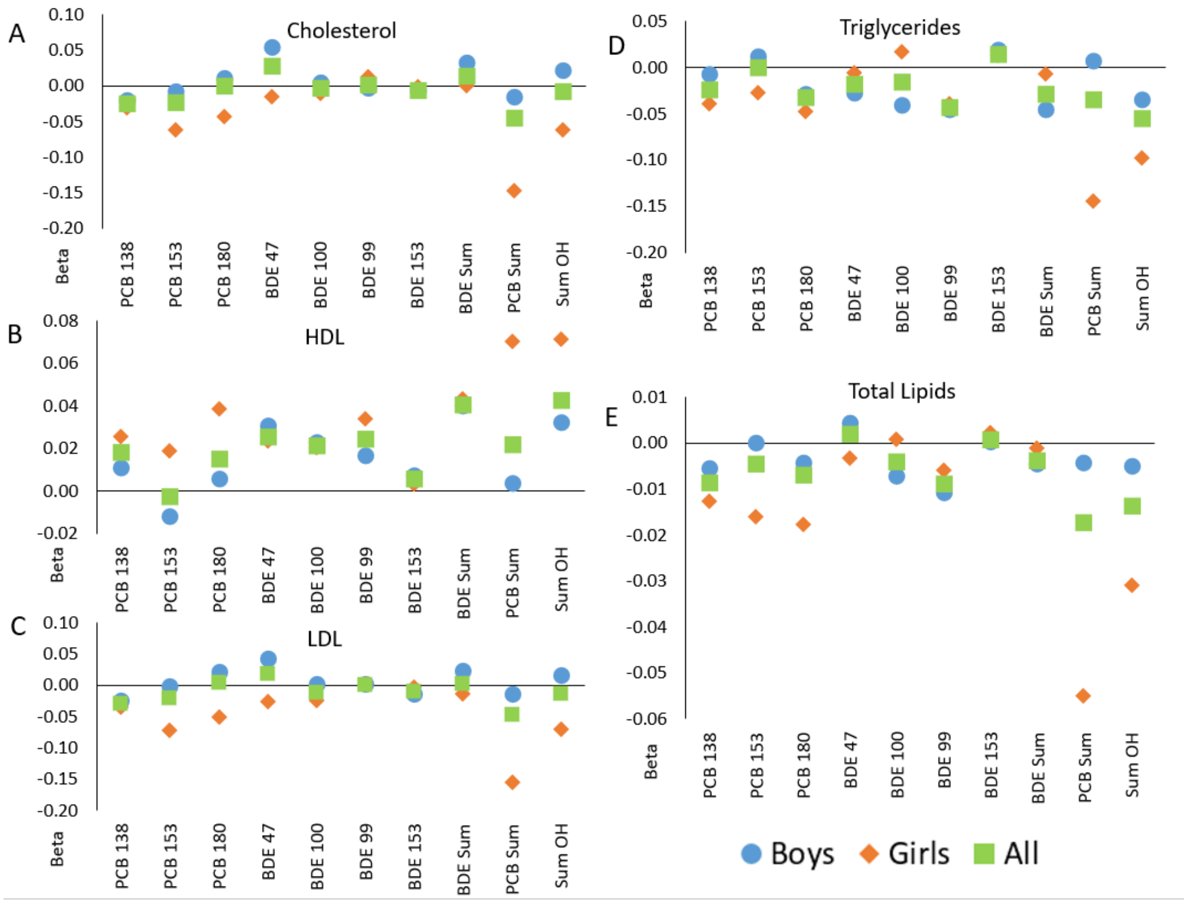


**Figure S1.** Correlations between POP and lipid levels in blood of 6–7 years old children in the GESTE cohort, overall and separate by child sex: A–total cholesterol; B–high density lipoproteins; C–low density lipoproteins, D–triglycerides; E–total lipids. Squares indicate results in the full cohort ($n$ = 147); offspring sex-specific estimates are shown with circles (in male offspring only, $n$ = 82) and diamonds (female offspring only, $n$ = 65). .
