# Supplementary material for: In Utero Exposure to Persistent Organic Pollutants and Childhood Lipid Levels
Source: Metabolites. 2021 Sep 28;11(10):657. doi: 10.3390/metabo11100657 (PMC8540619; doi:10.3390/metabo11100657)
Supplement: Supplementary file 1 [file metabolites-11-00657-s001.zip › Supplemental Tables_EI.pdf]

Table S1 - Pearson correlation coefficients and p-values for serum delivery and serum child organohalogens levels, GESTE Cohort.

			Child Chemicals				
			BDE 47	BDE 100	BDE 99	BDE 153	BDE 183
Delivery Chemicals	BDE 47	*r*	0.01	0.03	0.05	0.01	-0.04
		P	*0.95*	*0.68*	*0.57*	*0.87*	*0.61*
	BDE 100	*r*	-0.04	0.04	0.03	**0.24**	-0.04
		P	*0.67*	*0.64*	*0.76*	***0.005***	*0.62*
	BDE 99	*r*	-0.10	-0.08	-0.08	-0.01	-0.03
		P	*0.26*	*0.36*	*0.35*	*0.89*	*0.72*
	BDE 153	*r*	0.01	0.083	0.0752	**0.34**	-0.04
		P	*0.93*	*0.33*	*0.38*	***<.0001***	*0.66*
	PCB 138	*r*	-0.04	-0.024	-0.05	-0.03	-0.06
		P	*0.66*	*0.78*	*0.58*	*0.75*	*0.50*
	PCB 153	*r*	-0.06	-0.02	-0.06	-0.04	-0.08
		P	*0.51*	*0.78*	*0.47*	*0.65*	*0.35*
	PCB 180	*r*	0.04	0.10	0.02	0.12	-0.09
		P	*0.62*	*0.25*	*0.78*	*0.15*	*0.30*

Abbreviations: PCB - polychlorinated biphenyl, BDE - brominated diphenyl ether, r - Pearson correlation statistic, P - P-value from Pearson correlation

N for analyses is 140 child-mother pairs.

Table S2: Relationship between Chemical Exposures Levels at 6-7 years and Child Lipid Levels at 6-7 years in the GESTE Cohort.

		Bivariate Model			Multivariate Model[+]		
	Chemical	Beta	SE	P	Beta	SE	P
Cholesterol[a]	BDE 153	0.026	(0.043)	**	0.028	(0.043)	**
	BDE 47	0.027	(0.022)	**	0.031	(0.022)	**
	BDE 183	0.159	(0.087)	0.07	0.152	(0.087)	0.08
	BDE 100	0.024	(0.043)	**	0.024	(0.042)	**
	BDE 99	0.040	(0.029)	**	0.045	(0.029)	**
HDL[b]	BDE 153	-0.001	(0.001)	**	-0.001	(0.001)	**
	BDE 47	-0.030	(0.014)	0.04	-0.030	(0.014)	0.04
	BDE 183	0.040	(0.056)	**	0.038	(0.056)	**
	BDE 100	-0.061	(0.027)	0.02	-0.062	(0.027)	0.02
	BDE 99	-0.034	(0.018)	0.07	-0.035	(0.018)	0.06
LDL[a]	BDE 153	0.027	(0.042)	**	0.031	(0.042)	**
	BDE 47	0.040	(0.022)	0.07	0.044	(0.022)	0.04
	BDE 183	0.148	(0.086)	0.09	0.142	(0.085)	**
	BDE 100	0.039	(0.042)	**	0.041	(0.042)	**
	BDE 99	0.056	(0.028)	0.05	0.062	(0.028)	0.03
Triglycerides[b]	BDE 153	0.005	(0.031)	**	0.001	(0.031)	**
	BDE 47	0.015	(0.016)	**	0.014	(0.016)	**
	BDE 183	-0.016	(0.062)	**	-0.016	(0.062)	**
	BDE 100	0.044	(0.030)	**	0.041	(0.030)	**
	BDE 99	0.007	(0.021)	**	0.004	(0.021)	**
Total lipids[b]	BDE 153	0.004	(0.011)	**	0.004	(0.011)	**
	BDE 47	0.007	(0.006)	**	0.008	(0.005)	**
	BDE 183	0.028	(0.022)	**	0.026	(0.022)	**
	BDE 100	0.011	(0.011)	**	0.011	(0.011)	**

BDE 99	0.008	(0.007)	**	0.008	(0.007)	**

Abbreviations: SE - standard error, P - T-test P-value, HDL - high density lipoproteins, LDL - low density lipoproteins, PCB - polychlorinated biphenyl, BDE - brominated diphenyl ether, OH - organohalogens.

N=182

a Beta and SE from model using exposure untransformed and log transformed chemicals.

b Beta and SE from model using log transformed exposure and log transformed outcome.

** P-value from T-test > 0.10.

+ Adjusted for mother's age during pregnancy and mother's body mass index after delivery.
